# Facile fabrication of rice husk based silicon dioxide nanospheres loaded with silver nanoparticles as a rice antibacterial agent

**DOI:** 10.1038/srep21423

**Published:** 2016-02-18

**Authors:** Jianghu Cui, You Liang, Desong Yang, Yingliang Liu

**Affiliations:** 1 College of Materials and Energy, South China Agricultural University, Guangzhou 510642, Guang dong, P. R. China; 2 Guangdong Key Laboratory of Agricultural Environment Pollution Integrated Control, Guangdong Institute of Eco-Environmental and Soil Sciences, Guangzhou 510650, P. R. China; 3 College of Agriculture, Shihezi University, Shihezi 832000, Xinjiang, P. R. China

## Abstract

Bacterial leaf blight of rice caused by *Xanthomonas oryzae* pv. *oryzae* (*Xoo*) is a major disease of rice, leading to reduction in production by 10–50%. In order to control this disease, various chemical bactericides have been used. Wide and prolonged application of chemical bactericides resulted in the resistant strain of *Xoo* that was isolated from rice. To address this problem, we were searching for an environmentally friendly alternative to the commonly used chemical bactericides. In this work, we demonstrate that silicon dioxide nanospheres loaded with silver nanoparticles (SiO_2_-Ag) can be prepared by using rice husk as base material precursor. The results of the antibacterial tests showed that SiO_2_-Ag composites displayed antibacterial activity against *Xoo*. At cellular level, the cell wall/membrane was damaged and intercellular contents were leaked out by slow-releasing of silver ions from SiO_2_-Ag composites. At molecular level, this composite induced reactive oxygen species production and inhibited DNA replication. Based on the results above, we proposed the potential antibacterial mechanism of SiO_2_-Ag composites. Moreover, the cytotoxicity assay indicated that the composites showed mild toxicity with rice cells. Thus, this work provided a new strategy to develop biocide derived from residual biomass.

Rice (*Oryza sativa* L.) is an important cultivated food crop worldwide. It accounts for more than 20% of the world’s crop productions and feeds approximately 50% of the world’s population^1^. Bacterial blight, blast and sheath blight are the three most destructive diseases affecting rice^2^,^3^,^4^,^5^. Bacterial leaf blight is caused by *Xanthomonas oryzae* pv. *oryzae* (*Xoo*), and it can lead to a 10–50% reduction in crop yield. Various chemical bactericides have been used to control the infection and spread of *Xoo*, such as bismerthiazol and streptomycin^6^,^7^. Long-term and excessive exposure to the chemical bactericides have induced resistance in the bacteria, widespread instances of poor treatment efficacy and large economic losses^8^.

To solve this problem, scientists have been searching for several new antibacterial agents as alternatives to synthetic chemical bactericides. Various nanoparticles have been investigated for plant disease management, such as graphene oxide^9^,^10^, sulfur^11^, TiO_2_^12^, ZnO^13^, and Cu nanoparticles^14^. In particular, silver nanoparticles (Ag NPs) have attracted considerable attention due to their wide antibacterial spectrum compared to other nanomaterials^8^,^15^,^16^. Unfortunately, Ag NPs less than 20 nm in diameter tend to aggregate and oxidize, leading to a decrease in their antibacterial performance^17^,^18^,^19^. In addition, Ag NPs have been reported to have negative impacts or potential toxicities in plants^20^,^21^. Hence, there is a compelling need to improve the antibacterial activity and develop a valid strategy to reduce the toxicity of Ag NPs. Recent studies have shown that a wide range of base materials can be used to synthesize antibacterial composites with Ag NPs, including carbon nanotubes^22^, cellulose nanocrystals^23^ and graphene^24^. Our group also prepared some composites, such as carbon nanospheres^25^ and porous carbon^26^ loaded with Ag NPs. However, these materials require complex and expensive preparation methods. Therefore, the design of base materials with low cost and simple processing presents a significant challenge.

Rice husk is a commonly agricultural waste material and the world annual production is approximately 120 million tons^27^. It is reported that rice husk contains approximately 20% silicon dioxide, which makes it a potential renewable source of SiO_2_^28^. And previous researches have indicated that SiO_2_ nanoparticles can decrease heavy metal accumulation and increase the production of the plant^29^,^30^. Meanwhile, SiO_2_ nanospheres have high surface and reactivity and are considered as a supporting material according to the previous study^31^. So we use rice husk as a raw material to prepare SiO_2_ nanosphere, which is a suitable candidates for supporting material.

In our work, we developed a simple method to synthesize SiO_2_ nanospheres using rice husk as a raw material and decorated the nanospheres with Ag NPs by the reduction of Ag ions. The results showed that the Ag NPs were successfully loaded onto the surface of the SiO_2_ nanospheres (SiO_2_-Ag), and the preliminary studies showed that the composites have a superior antibacterial effect and only mild cytotoxicity to rice cells. Furthermore, the level of reactive oxygen species (ROS), the content of genomic DNA and the integrity of the cell membrane were also determined. Based on the results above, we proposed a potential antibacterial mechanism of the SiO_2_-Ag composites. In general, this study provides direct evidence that these composites have great potential to be used as antibacterial agents in agriculture and offer an environmentally friendly method to synthesize an antibacterial nanomaterial using residual biomass.

## Results and Discussion

### Synthesis and characterization of the SiO_2_-Ag composites

**Figure 1:**
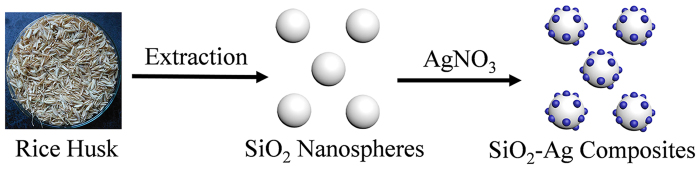
Schematic illustration of the formation of Ag NPs immobilized on SiO_2_ nanospheres. White sphere and blue points stand for SiO_2_ nanospheres and Ag NPs, respectively.

**Figure 2:**
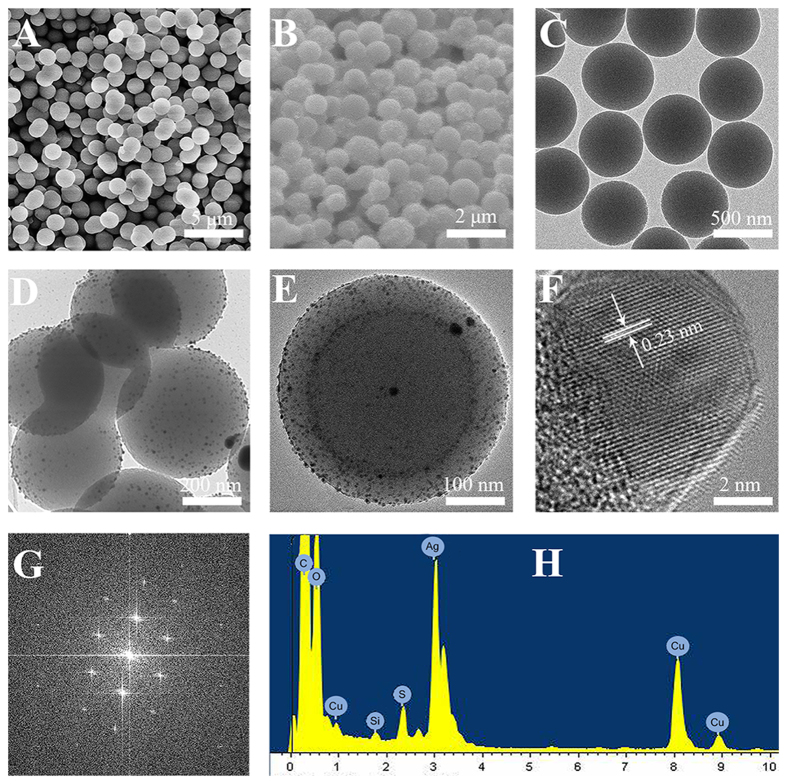
The characterization of SiO_2_ nanospheres and SiO_2_-Ag composites. Scanning electron microscopy (SEM) images of SiO_2_ nanospheres (**A**), SiO_2_-Ag composites (**B**); Transmission electron microscope (TEM) images of SiO_2_ nanospheres (**C**) and SiO_2_-Ag composites (**D**); HRTEM images of SiO_2_-Ag composites (**E**) and Ag NPs (**F**); Fast fourier transform images of Ag NPs (**G**); EDS of SiO_2_-Ag composites (**H**).

The synthesis process for the SiO_2_-Ag composites is shown in Fig. 1. In this study, we used rice husks as a raw material to synthesize SiO_2_ nanomaterials by a hydrothermal method. SiO_2_ was a near-perfect sphere with a smooth surface and a diameter of approximately 400 nm (Fig. 2A,C). Then, poly-(N-vinyl-2-pyrrolidone) (PVP) was applied as the stabilizer and reductant. The Ag ions were reduced at the surface of SiO_2_ nanospheres, and the typical morphology of the SiO_2_-Ag composites is shown in Fig. 2B,D,E. We propose that the bulges on the SiO_2_ nanospheres are Ag NPs.

We performed high- resolution TEM (HRTEM) (Fig. 2E,F) to further analyze the nanostructures of the SiO_2_-Ag composites and found that the diameter of the Ag NPs was approximately 10 nm. This small size of the Ag NPs might have better antibacterial activity^32^,^33^. The interplanar spacing for the lattice fringes was approximately 0.23 nm, corresponding to the (111) lattice plane of silver^34^,^35^. The elemental composition of the SiO_2_-Ag composites was analyzed by energy dispersive X-ray spectroscopy (EDS), as shown in Fig. 2H; several types of peaks were clearly observed, which correspond to carbon, oxygen, copper, silicon, and silver. The as-prepared SiO_2_-Ag composites contain approximately 57.26 wt% Si and 5.47 wt% Ag.

**Figure 3:**
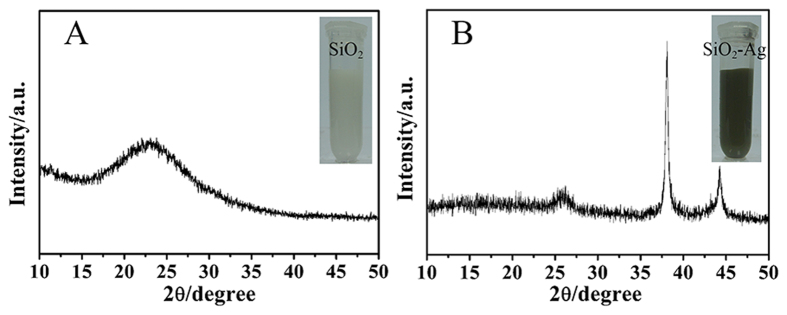
XRD patterns of SiO_2_ nanospheres (**A**) and SiO_2_-Ag composites (**B**).

The structural features of the SiO_2_-Ag composites have also been investigated by X-ray diffraction (XRD) analysis (Fig. 3). As shown in Fig. 3A, the typical XRD pattern of SiO_2_ nanospheres was an amorphous peak with the equivalent Bragg angle at 2θ = 22°. Figure 3B indicates that the XRD pattern of the SiO_2_-Ag composites has two sharp Bragg peaks at 38.2°, 44.4° in the 2θ region, which could be assigned to the (111) and (200) planes of silver, indicating that Ag NPs were successfully loaded onto the surface of the SiO_2_ nanospheres.

### Release property

**Figure 4:**
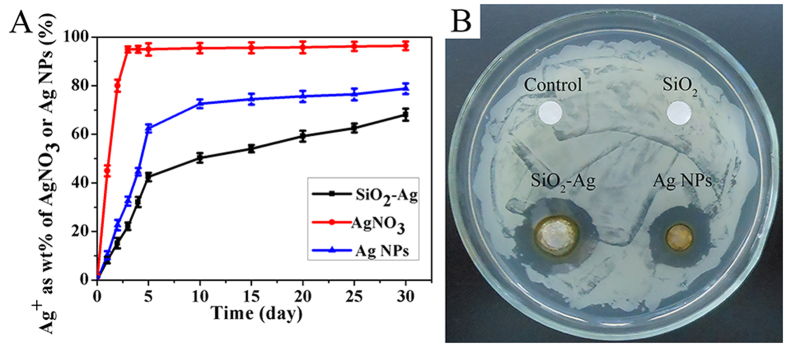
(**A**) Release of Ag ions from AgNO_3_, Ag NPs and SiO_2_-Ag composites at 37 °C. (**B**) Zone of inhibition for control, SiO_2_ nanospheres, Ag NPs and SiO_2_-Ag composites against *Xoo*.

An inductively multitype coupled plasma emission spectrometer (ICP-AES) was used to compare the release rate of Ag ions from AgNO_3_, Ag NPs and the SiO_2_-Ag composites. As shown in Fig. 4A, the Ag ions were completely released into the ultrapure water from AgNO_3_ and the Ag NPs in less than 3–10 d. However, the SiO_2_-Ag composites could stably release Ag ions over 30 d. The Ag ion release rate of the composites was slower than that of AgNO_3_ and the Ag NPs. After 30 d, the SiO_2_-Ag composites had released 68.1% of the Ag ions, which was significantly lower than AgNO_3_ (96.4%) and the Ag NPs (78.8%). The results showed that the SiO_2_-Ag composites have a lower release speed than AgNO_3_ and the Ag NPs, which had more long-term antibacterial effects than that of AgNO_3_ and Ag NPs^36^,^37^.

### Antibacterial evaluation

**Figure 5:**
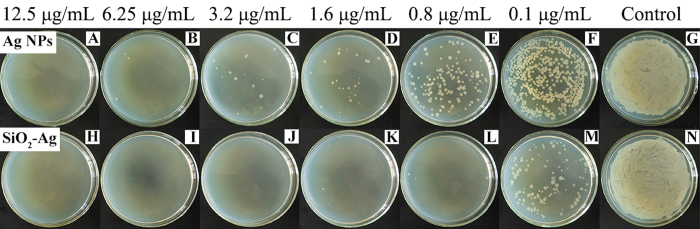
MIC of Ag NPs and SiO_2_-Ag composites against *Xoo*. The concentration of the antibacterial agents is shown on the top of each plate.

To confirm the antibacterial effect of the SiO_2_-Ag composites, the growth inhibition of the tested bacteria *Xoo* was investigated by the disk diffusion method. As shown in Fig. 4B, Ag NPs and SiO_2_-Ag composites have an average diameter of the inhibition zone of 18 ± 2 mm and 23 ± 1 mm, respectively. However, the disks with the control and SiO_2_ nanospheres have no inhibition zone, indicating that the Ag NPs are the effective antibacterial component of the SiO_2_-Ag composites. Minimum inhibitory concentration (MIC) testing against *Xoo* was carried out to further evaluate the antibacterial activity of the SiO_2_-Ag composites. As shown in Fig. 5, the density of bacterial growth was decreased in a dose-dependent manner. *Xoo* growth was completely inhibited when the concentration of the SiO_2_-Ag composites was 3.2 μg/mL (Fig. 5J), whereas the Ag NP solution exhibited the same effect at a concentration of 12.5 μg/mL (Fig. 5A). The tests of the antibacterial properties confirmed that the antibacterial activity of the SiO_2_-Ag composites was approximately four times higher than that of the Ag NPs against *Xoo*.

**Figure 6:**
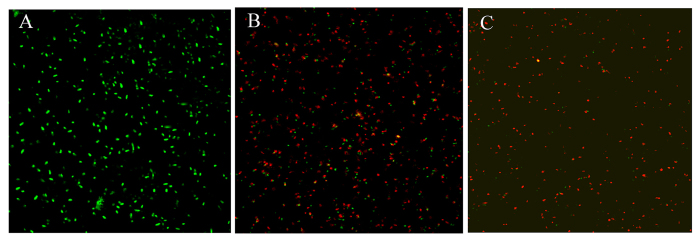
Confocal fluorescent microscopy images of live and dead *Xoo* cells after incubation with different samples. Fluorescence image of *Xoo* treated with control (**A**); Fluorescence image of *Xoo* treated with Ag NPs (**B**); Fluorescence image of *Xoo* treated with SiO_2_-Ag composites (**C**). Green spots represent live bacterial cells, whereas red fluorescence indicates dead bacteria.

The ability of SiO_2_-Ag to prevent viable bacteria growth is also demonstrated by fluorescence staining. Ethidium bromide (EB) and acridine orange (AO) were used as live/dead coloring agents. EB could enter through the damaged cell membrane and selectively stain dead cells, whereas AO could penetrate live and dead cells^25^,^38^. As shown in Fig. 6, nearly all of bacteria were viable when cultured on the control and SiO_2_-Ag composites. In contrast, the *Xoo* treated with the SiO_2_-Ag composites exhibited strong red fluorescence, indicating that most of the bacteria were killed (Fig. 6C). These results further support the antibacterial studies that the SiO_2_-Ag composites were clearly more effective than the Ag NPs.

### Cell wall/membrane integrity assay

**Figure 7:**
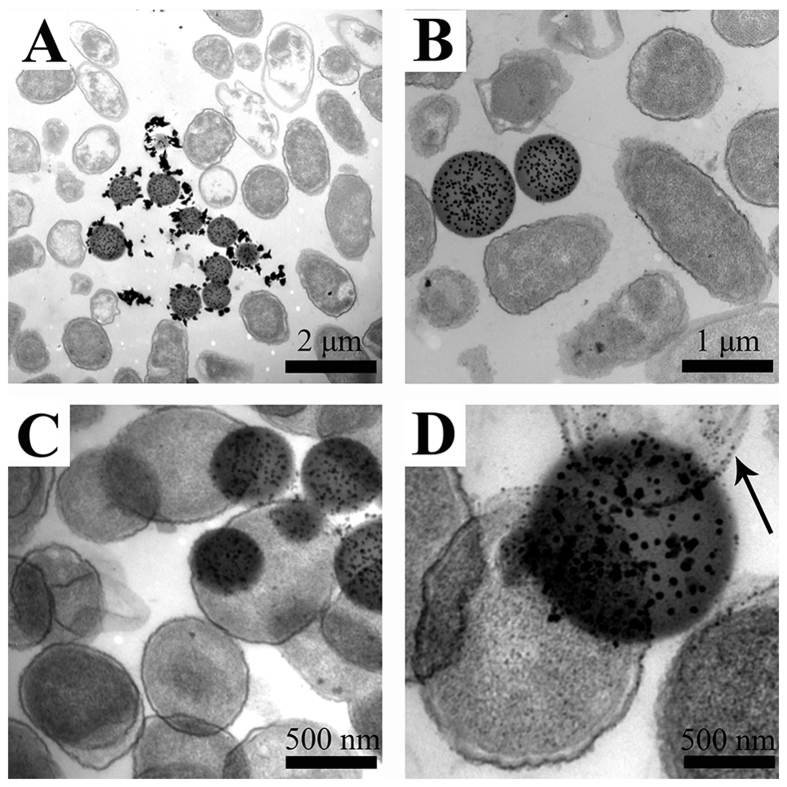
Typical TEM images of *Xoo* treated with SiO_2_-Ag composites.

A TEM study was performed to observe the morphological changes of bacteria cells after treatment with the SiO_2_-Ag composites. As shown in Fig. 7A,B, the bacteria were adsorbed by the SiO_2_-Ag composites, and the morphology of bacteria cells changed from cylindrical to spherical after treatment with the SiO_2_-Ag composites for 2 h. Figure 7C,D illustrate that released Ag ions disrupted the cell wall/membrane integrity. As a result, more Ag NPs were internalized into the bacteria cell wall/membrane, and the contents of the cell leaked out, leading to protein denaturation and cell death.

The antibacterial results demonstrate that the SiO_2_-Ag composites have better antibacterial properties compared to those of the Ag NPs. According to the literature, the antibacterial activity of Ag NPs would be reduced due to aggregation and oxidation^17^,^18^,^19^. In our work, we prepared the composites such that the Ag NPs were loaded on the surface of the SiO_2_ nanospheres. These composites could effectively enhance the antibacterial activity by preventing the aggregation and oxidation of Ag NPs and by continuously releasing Ag ions. This result was consistent with previous studies^25^,^39^. The SiO_2_-Ag composites have a large surface area and high adsorption properties; thus, the bacteria could be easily adsorbed by the composites.

### Intracellular oxidative stress

**Figure 8:**
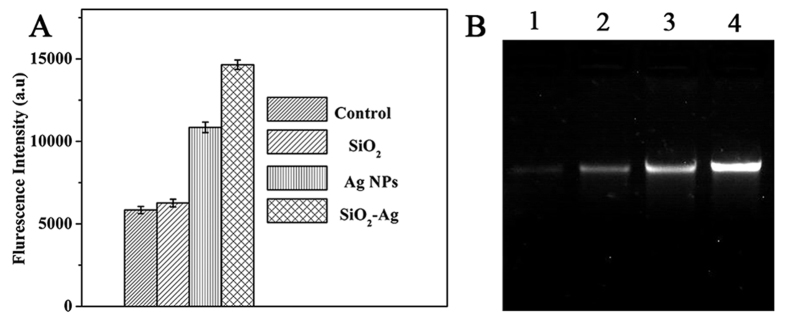
(**A**) Formation of ROS in *Xoo* cells after a 2 h incubation period with the control, SiO_2_ nanospheres, Ag NPs and the SiO_2_-Ag composites. ROS were detected by fluorescence measurement of the reporter DCF. Each data point represents the mean value from at least three independent experiments. (**B**) Electrophoresis analysis of genomic DNA in *Xoo* cells treated with different concentrations of the SiO_2_-Ag composites for 4 h. Lanes 1, 2, 3 and 4 represent genomic DNA extracted from *Xoo* cells treated with 12.5, 3.2, 0.8 and 0 μg/mL of the SiO_2_-Ag composites, respectively.

It has been suggested that the production of ROS is the common pathway by which antibacterial agents induce oxidative damage in bacteria cells^40^. Many nanomaterials have been reported to exert their toxic effects through ROS^41^,^42^,^43^. Therefore, we compared the level of ROS after treatment with SiO_2_ nanospheres, Ag NPs and the SiO_2_-Ag composites by fluorescence intensity. As shown in Fig. 8A, the DCF fluorescence intensity in samples treated with SiO_2_ nanospheres was similar to that in the untreated cells. However, in the presence of the Ag NPs, the DCF fluorescence intensity was increased two-fold compared with exposure to the SiO_2_ nanospheres and to untreated cells. In addition, the DCF fluorescence intensity of the samples treated with the SiO_2_-Ag composites was nearly 1.4 times higher than that of the samples treated with Ag NPs. These results revealed that the SiO_2_-Ag composites could significantly increase ROS production and lead to cell damage. Moreover, the SiO_2_-Ag composites would have a long-term antibacterial effect by continually releasing Ag ions.

### Influence of SiO_2_-Ag composites on genomic DNA

**Figure 9:**
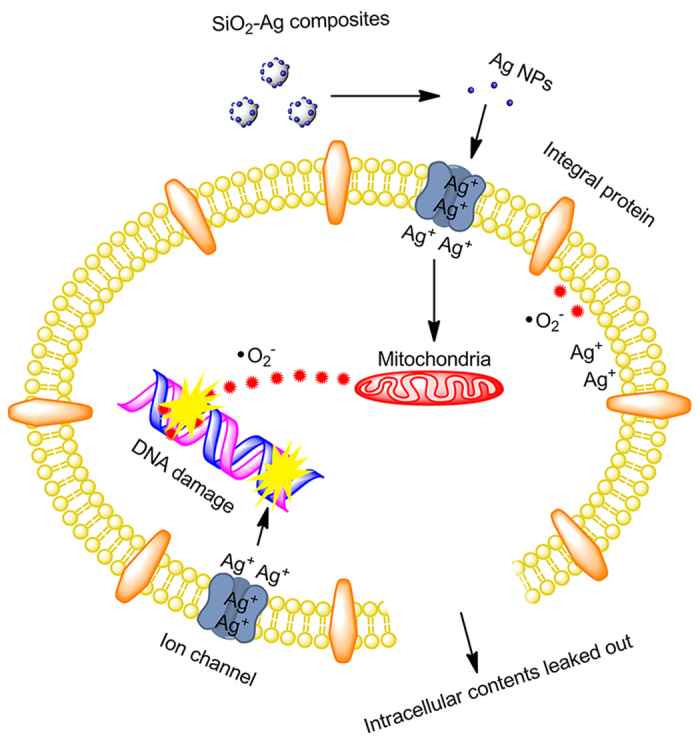
Schematic of the antibacterial mechanism of the SiO_2_-Ag composites.

It has been reported that Ag NPs interact with DNA and inhibit DNA replication, resulting in rapid antibacterial activity^44^,^45^. In this study, agarose gel electrophoresis analysis was used to investigate the possible antibacterial mechanism of the SiO_2_-Ag composites. As shown in Fig. 8B, the intensity of the genomic DNA band was decreased in a dose-dependent manner. The intensity of the genomic DNA band was lowest when the cells were treated with 12.5 μg/mL of SiO_2_-Ag composites. In contrast, the intensity of the genomic DNA band of the untreated cells formed a clear band. The results of agarose gel electrophoresis analysis were consistent with the MIC values. According to the above results, the potential antibacterial mechanism of the SiO_2_-Ag composites was proposed as follows (Fig. 9): the bacterial cells absorb on the surface of the SiO_2_-Ag composites by electrostatic forces, and Ag ions were released from the Ag NPs and transported to the cytoplasm. The Ag ions directly interact with intracellular mitochondria, resulting in the generation of ROS and the inhibition of DNA replication. Subsequently, the integrity of the cell wall/membrane was disrupted, and the intracellular contents leaked out.

### Cytotoxicity assay

**Figure 10:**
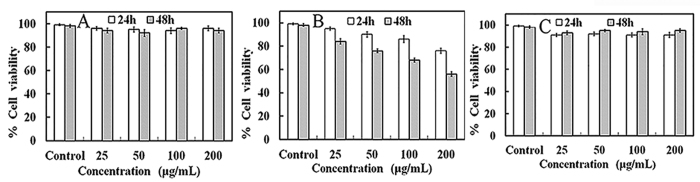
Cell viability assay for *Xoo* cells. Influence of different concentrations of SiO_2_ (**A**), Ag NPs (**B**) and SiO_2_-Ag composites (**C**) on rice cell viability. Each data point represents the mean value from at least three independent experiments.

To test the toxicity of the SiO_2_-Ag composites, we selected rice cell viability to elucidate the cellular response to a toxin. The rice cell suspension was exposed to different concentrations of SiO_2_ nanospheres, Ag NPs or the SiO_2_-Ag composites for 24 or 48 h (Fig. 10). The results showed that the cell viability with Ag NPs was lower than that of the SiO_2_ nanospheres and SiO_2_-Ag composites after a 24 h culture. However, after culturing for 48 h, the viability of cells treated with Ag NPs was significantly lower than that of the SiO_2_ nanospheres and SiO_2_-Ag composites. Increasing the incubation time and concentration of the Ag NPs resulted in a significant decrease in cell viability. In our study, the different results between the *Xoo* and rice cells might have occurred because the structure of bacteria and plant cells is different and the content of silver in the SiO_2_-Ag composites was low and had no impact on the rice cells. More detailed reasons require further study. Therefore, we anticipate that the SiO_2_-Ag composites are promising antibacterial agents that would control rice diseases effectively and provide rice plants with nutrients.

In summary, we have developed a simple and environmentally friendly method using rice husks as a raw material to synthesize SiO_2_ nanospheres. These materials were then decorated with Ag NPs by the reduction of Ag ions in the presence of PVP as a stabilizer and reductant. TEM, SEM and XRD indicated that Ag NPs with small sizes were well dispersed onto the surface of SiO_2_ nanospheres. This structure could prevent the aggregation and oxidation of Ag NPs. We also confirmed that the SiO_2_-Ag composites displayed antibacterial activity against *Xoo* that was approximately four times higher than that of the Ag NPs. Meanwhile, the antibacterial mechanism of the SiO_2_-Ag composites was explored. The Ag ions released from the SiO_2_-Ag composites could induce the production of ROS, leading to the inhibition of DNA replication and disruption of the cell wall/membrane. More importantly, the cytotoxicity assay indicated that the SiO_2_-Ag composites showed only mild toxicity towards rice cells. Thus, the SiO_2_-Ag composites have a great potential application in rice disease management as antibacterial agents. This work provides a new strategy of environmentally friendly synthesis of antibacterial nanomaterials using residual biomass.

## Methods

### Synthesis of SiO_2_ nanospheres

SiO_2_ nanospheres were synthesized using rice husks. The rice husks were washed with distilled water and milled into powder. Next, 2.0 M NaOH was mixed with the rice husk powder at a ratio of 1:7 (w/v) in a 200 mL three-neck round-bottom flask equipped with a thermometer and heated to 100 °C for 4 h. The extract was separated from the solids by vacuum-assisted filtration and diluted with different volume ratios of distilled water and ethanol at 25 °C. Then, sulfuric acid (1.0 M) was added drop-wise into the system until the pH of the system was approximately 9.0. Using an ultrasonicator, polyethylene glycol was completely dissolved into the solution, followed by the drop-wise addition of a solution of 1 M sulfuric acid to lower the pH to 3. The mixture was left standing for 10 min at 25 °C and then centrifuged for 5 min. The products were washed several times with distilled water and dried at 60 °C for 5 h. To obtain the SiO_2_ nanospheres, the samples were calcined at 550 °C for 1 h in a muffle furnace to remove residual organics in the SiO_2_ nanosphere sample.

### Synthesis of SiO_2_-Ag composites

To form a homogeneous SiO_2_-Ag composite suspension, 100 mg of the SiO_2_ nanosphere powder in 100 mL of deionized water was sonicated for 30 min, and 6 g PVP was dissolved in the previous solution. Then, 20 mL of an AgNO_3_ (1 mM) aqueous solution was rapidly added into the above solution. This mixture was stirred vigorously for 12 h in the dark at 80 °C. The resulting product was collected by centrifugation at 5,000 rpm for 10 min and further washed in deionized water several times to remove residual Ag ions. The dry SiO_2_-Ag composites were obtained after drying under vacuum for 3 h at 60 °C.

### Characterization

The obtained samples were characterized by XRD performed on a X-ray diffractometer with a Cu target in the 2θ range from 5° to 80° (40 kV, 30 mA, λ = 1.54051 Å). The surface morphologies of the samples were characterized by FEI Quanta 200 SEM. Morphological features of the SiO_2_-Ag composites were observed by a Philips TECNAI 10 TEM and a JEOL JEM-2100F field emission electron microscope equipped with Oxford INCA Energy TEM 200 EDS devices with an accelerating voltage of 200 kV. The concentration of silver was estimated using ICP-AES.

### Release property

The concentrations of Ag ions released from the samples were monitored by ICP-AES. Approximately 1 mg of Ag NPs, the SiO_2_-Ag composite solid and the same equivalent amount of AgNO_3_ were immersed in 5 mL of ultrapure water on a slow shaking incubator at 37 °C. After 1 d, the concentrations of Ag ions were measured, and the solutions were transferred into dialysis tubes and immersed in 100 mL of ultrapure water every 5 d. The concentration of Ag ions in the ultrapure water solutions was measured by ICP-AES.

### Antibacterial activity

Before the experiment, petri dishes and solid Luria-Bertani (LB) medium must be sterilized in an autoclave at 121 °C for 20 min. The antibacterial properties of the Ag NPs and SiO_2_-Ag composites were evaluated by testing the disk diffusion inhibitory zone and minimum inhibitory concentration breakpoints.

#### Disk diffusion inhibition zone

In this test, we adjusted the concentration of the bacterial suspension until the optical density was approximately 0.1 at 600 nm. The concentrations of Ag NPs and SiO_2_-Ag composites were 200 μg/mL. The standard small piece of filter paper (6 mm) containing SiO_2_ nanospheres, Ag NPs and SiO_2_-Ag composites were placed onto LB agar plates swabbed with bacteria. The plates were incubated for 16 h, and the zone of inhibition of bacterial growth was used as a measurement of susceptibility. Sensitivity was inversely proportional to the diameter of the zone.

#### Minimum inhibitory concentration (MIC)

The MIC was determined by the lowest concentrations of Ag NPs and SiO_2_-Ag composites that restrain bacteria growth after overnight incubation. First, the bacteria were incubated at 37 °C for 12 h in LB liquid medium, and the bacterial solution was diluted with LB to an optical density of 0.1 at 600 nm. The Ag NPs and SiO_2_-Ag composite solutions with different concentrations (0, 0.05, 0.1, 0.2, 0.4, 0.8, 1.6, 3.2, 6.25, 12.5, 25, 50, 100 and 200 μg/mL) were prepared. Then, the bacterial solution (100 μL) and 0.9 mL of LB liquid medium were added to 1 mL of each of the different concentrations of Ag NPs and SiO_2_-Ag composite solutions, and these solutions were placed onto a rotary shaker at 120 rpm at 37 °C for 2 h. After that, 100 μL of the bacterial solution was added to each agar plate and spread evenly. Later on, all of the agar plates were sealed with parafilm and incubated at 37 °C for 18 h. Finally, the MIC was determined by the lowest concentrations of Ag NPs and SiO_2_-Ag composites that inhibited the visible growth of bacteria^46^.

#### Fluorescence imaging

Bacteria were incubated in the LB liquid medium supplemented with SiO_2_ nanospheres, Ag NPs and the SiO_2_-Ag composites. The bacterial cells were collected by centrifugation and washed three times with phosphate buffer saline (PBS) and then stained using EB and AO for 15 min. After washing with PBS, the samples were observed by fluorescence microscopy.

### Cell morphological change

To observe the morphological changes of bacterial cells after treatment with the SiO_2_-Ag composites, the *Xoo* cells were exposed to the SiO_2_-Ag composites (12.5 μg/mL) in microtiter plates with silicon chips in the bottom. After the cultures grew for 2 h, the silicon chip was harvested and processed for TEM. First, the silicon chip was removed from the microtiter plates and washed three times with buffer. Then, the samples were fixed in 2.5% glutaraldehyde for 2 h. After fixation, the silicon chip was rinsed with buffer twice. The samples were washed with a cacodylate buffer and fixed in 1% osmium tetraoxide. Then, sample embedding was carried out using a standard protocol. The slices were deposited on bare #200 mesh grids and stained with uranyl acetate for 5 min. Finally, the grids were dried in a desiccator and examined using TEM.

### Intracellular reactive oxygen species measurement

2,7-dichlorofluoroscein diacetate (DCFH-DA) was used to further identify the intracellular generation of ROS in the treated bacterial cells. The DCFH-DA could enter the cell and react with ROS, which formed the highly fluorescent compound dichlorofluorescein (DCF). Experimental procedures were followed as described previously^41^,^47^. The fluorescent signal intensity of DCF (with an emission wavelength of 525 nm) was recorded using a fluorescence spectrophotometer with an excitation wavelength of 488 nm.

### Genomic DNA isolation

The genomic DNA was extracted from the *Xoo* cells treated with different concentrations of SiO_2_-Ag composite (12.5, 3.2, 0.8 μg/mL) for 4 h. Then, the DNA was isolated by the phenol chloroform extraction method^48^. The isolated DNA was then analyzed on a 1% agarose gel using EB.

### Cytotoxicity assay

Rice suspension cells were cultured according to the literature procedure^49^. The seeds were surface-sterilized in 75% ethanol for 1 min and 0.1% mercury chloride for 10 min and rinsed five times with sterile distilled water. The seeds were incubated in plastic petri dishes containing modified N6 medium. The sealed dishes were cultured in the dark to induce calli from mature rice seeds at 28 °C. Every 7 d, the calli were subcultured in the subculture medium. After 4 weeks, the calli were transferred to 125 mL conical flasks containing 40 mL of liquid AA medium and placed on a rotary shaker at 110 rpm at 28 °C in the dark. To supplement nutrients, the suspension cells were subcultured at 5 d intervals for 2–3 months by replacing the old nutrient solution medium every 5 d.

Then, the culture medium was replaced with 100 μL of different concentrations of SiO_2_ nanospheres, Ag NPs or the SiO_2_-Ag composites. The cells were further incubated for 24 or 48 h, and then, 25 μL of MTT (3-(4,5-dimethyl-2-yl)-2,5-diphenyl tetrazolium bromide) (5 mg/mL) was added to each culture medium until the final concentration was 1 μg/mL. After incubation for another 2 h, the absorbance was measured at 570 nm using a microplate reader. Cell viability was normalized to that of rice cells cultured in the cell media. Measurements were repeated three times for each concentration.

## Additional Information

**How to cite this article**: Cui, J. *et al*. Facile fabrication of rice husk based silicon dioxide nanospheres loaded with silver nanoparticles as a rice antibacterial agent. *Sci. Rep.* **6**, 21423; doi: 10.1038/srep21423 (2016).
